# Sensitivity of Planktonic Cells of *Staphylococcus aureus* to Elevated Hydrostatic Pressure as Affected by Mild Heat, Carvacrol, Nisin, and Caprylic Acid

**DOI:** 10.3390/ijerph17197033

**Published:** 2020-09-25

**Authors:** Jyothi George, Sadiye Aras, Md Niamul Kabir, Sabrina Wadood, Shahid Chowdhury, Aliyar Cyrus Fouladkhah

**Affiliations:** 1 Public Health Microbiology Laboratory, Tennessee State University, Nashville, TN 37209, USA; jgeorge3@tnstate.edu (J.G.); saras@my.tnstate.edu (S.A.); mkabir@tnstate.edu (M.N.K.); swadood@tnstate.edu (S.W.); schowdh1@tnstate.edu (S.C.); 2 Department of Biological Sciences, Tennessee State University, Nashville, TN 37209, USA; 3 Cooperative Extension Program, Tennessee State University, Nashville, TN 37209, USA

**Keywords:** *Staphylococcus aureus*, high-pressure processing, carvacrol, nisin, caprylic acid

## Abstract

Current study investigated effects of elevated hydrostatic pressure exposure in the presence of mild heat and natural antimicrobials against *Staphylococcus aureus*. Hydrostatic pressure of 350 to 550 MPa with nisin (5000 IU/mL), carvacrol, or caprylic acid (0.5% *v*/*v*) were applied for the reduction in four-strain mixture of *S. aureus* in HEPES buffer at 4 and 40 °C for up to 7 min. Results were statistically analyzed by ANOVA and D-values were additionally calculated using best-fitted linear model. Prior to exposure to treatments at 4 °C, counts of the pathogen were 7.95 ± 0.4 log CFU/mL and were reduced (*p* < 0.05) to 6.44 ± 0.3 log CFU/mL after 7 min of treatment at 450 MPa. D-value associated with this treatment was 5.34 min (R^2^ = 0.72). At 40 °C, counts were 8.21 ± 0.7 and 5.77 ± 0.3 log CFU/mL before and after the 7-min treatments, respectively. D-value associated with 40 °C treatment was 3.30 min (R^2^ = 0.62). Application of the antimicrobials provided additional pathogen reduction augmentation for treatments < 5 min. The results of the current study could be incorporated for meeting regulatory requirements such as Food Code, HACCP, and Preventive Control for Human Food of Food Safety Modernization Act for assuring microbiological safety of products against this prevalent pathogen of public health concern.

## 1. Introduction

Infections caused by foodborne pathogens of public health concern are persisting challenges in healthcare settings, for the food industry, and for the consumers of raw and processed commodities. The symptoms of foodborne diseases range from mild and self-limiting manifestations such as nausea, vomiting, and diarrhea to severe complications such as kidney and liver failure, and brain and neural disorders that could result in absence from work, potential life-long health complications, and premature death [1]. At least, 31 known foodborne pathogens are estimated to cause 9.4 million illnesses, 56,961 hospitalizations, and 1351 death episodes in a typical year in the United States [2]. Foodborne diseases are an important cause of morbidity and mortality around the world as well. Around 600 million foodborne disease episodes and 420,000 deaths are associated with foodborne diseases globally every year with 30% of deaths occurring among the children under the age of 5 [1]. These challenges are expected to be augmented in the landscape of climate change as increases in environmental temperature could augment the proliferation and persistence of an array of microbial pathogens in food and water supplies [3,4,5,6].

Among these foodborne pathogens, *Staphylococcus aureus* is a commensal bacterium and can potentially be isolated from the mucosa or skin of both humans and animals [7]. Some studies indicate that *S. aureus* could be isolated from >20% of food processing employees [8,9]. As such, food handlers and food products are considered one of the main reservoirs of dissemination of this pathogen in the community [10]. Epidemiological data obtained from the U.S. Centers for Disease Control and Prevention indicate that from 1998 to 2017 there were more than 600 outbreaks (single or multi-state events) in the United States leading to around 10,000 cases of illness episodes associated with *S. aureus* [11].

Among the various methods of food preservation, the application of high-pressure processing is gaining increasing importance and momentum in food commerce. The technology exposes the final packaged products to elevated levels of pressure, typically from 100 to 1000 MPa (most commonly around 650 MPa) for treatments commonly lasting less than 10 min [12,13,14]. The National Advisory Committee on Microbiological Criteria for Foods (NACMCF) has defined high-pressure pasteurization as a “pasteurization equivalence,” thus an array of products is currently available in the market relying on this emerging technology for microbiological safety [15]. One of the main challenges associated with this technology is the slightly higher operation costs of pressure-treated products relative to traditional heat-treated commodities [16]. The application of treatment at elevated pressures of 650 MPa (currently the most common intensity in food commerce) and higher intensity levels is typically associated with the increased operation and maintenance cost of pressure vessels. Thus, the application of mild pressure in combination with natural antimicrobials could be of importance for the stakeholders of this technology. The application of mild levels coupled with natural antimicrobials, if proven to be microbiologically efficacious, could have additional co-benefits such as the potential for better retention of nutrients, increased shelf-life due to residual effects of antimicrobials, and better organoleptic properties [12,16].

Various antimicrobials in the United States are regulated by the generally recognized as safe (GRAS) list of the U.S. Food and Drug Administration [17]. Although the efficacy of antimicrobials from GRAS list has been extensively studied in the literature, limited studies are available exploring the efficacy of these antimicrobials for augmenting the performance of emerging and non-thermal processes, particularly their antimicrobial effectiveness under elevated hydrostatic pressure [14]. Nisin is a peptide with antimicrobial properties that is produced by bacterium *Lactococcus lactis* and is the first bacteriocin approved for commercial utilization in food industry. Various concentrations of this chemical, including 1000 to > 5000 IU, have been studied as an antimicrobial agent against an array of microorganisms, particularly Gram-positive bacteria [18,19,20,21,22,23]. Caprylic acid (C8H16O2), found primarily in bovine milk, extracted palm and coconut oil, and carvacrol (C10H14O), found primarily in oregano, both belong to the GRAS list as well. These bioactive compounds with antimicrobial properties have been utilized in the food industry to assure the microbiological safety and extend the shelf-life of various products [24,25,26,27,28]. 

Considering the public health importance of *S. aureus* and its prevalence in food products and food manufacturing facilities, the current study investigates the effects of elevated hydrostatic pressure for decontamination of planktonic cells of the pathogen. This study further investigates the effects of natural antimicrobials (nisin, caprylic acid, and carvacrol) for augmenting the decontamination efficacy of pressure-based pasteurization. 

## 2. Materials and Methods

### 2.1. Propagation of Bacterial Cell and Inoculation Mixture

This study utilized a four-strain mixture of *S. aureus* from the strain library of the Public Health Microbiology laboratory in Nashville, TN. These strains were selected based on their epidemiological and food industry significance and were originally obtained from American Type Culture Collection (ATCC^®^, Manassas, VA, USA) with strain designation numbers of ATCC^®^ 51740^TM^, ATCC^®^ 6538^TM^, ATCC^®^ 25923^TM^, and ATCC^®^ 9144^TM^. The glycerol stock of these strains were kept at −80 °C and prior to the experiment, 100 µL of each strain was aseptically pipetted into 10 mL of Tryptic Soy Broth (Difco, Becton Dickinson, Franklin Lakes, NJ, USA), supplemented with 0.6% yeast extract (TSB + YE), to activate each strain. The addition of yeast extract was designed to minimize the acid-stress of the pathogen during culturing [12]. The inoculated TSB + YE was then aerobically incubated at 37 °C for 22–24 h, and after homogenizing the overnight bacterial suspension by a high-speed vortex (Scientific Industries, Model Vortex-2 Genie, Bohemia, NY, USA), a 100 µL aliquot of the homogenized bacterial suspension, for each strain separately, was transferred to new, sterile, 10-mL TSB + YE. In order to prepare the sub-cultured bacterial suspension, the inoculated TSB + YE was again aerobically incubated at 37 °C for 22–24 h. The bacterial cells from the sub-cultured suspensions were then harvested, for each strain individually, as elaborated in our recent studies [12,13,14,16]. In short, bacterial cells (2 mL for each strain) after homogenizing the overnight suspension by vortexing, were harvested by centrifugation (Eppendorf North America, Hauppauge, NY, USA) for 15 min at 6000 RPM (equivalent to approximately 3540 g, for the centrifuge’s 88 mm rotor [Model 5424, Rotor FA-45-24-11]). The cells, for each strain separately, were then resuspended in 2 mL of HEPES buffer (VWR International Inc., Suwanee, GA, USA). This purification step was repeated twice using the same intensity and time and the pellets were resuspended in HEPES buffer. Individually activated, cultured, sub-cultured, and purified strains were then composited before each experiment to assure equal representation of four strains prior to treatments.

### 2.2. High-Pressure Processing and Antimicrobial Treatments

The current study utilized the application of three antimicrobials with concentrations relevant to the food industry. The concentrations and the pressure intensity levels were selected based on preliminary trials and our recent studies [13,14]. A powder form of nisin (Sigma-Aldrich, St. Louis, MO, USA) was used for preparation of the antimicrobial. This antimicrobial, generally considered as safe by the U.S. Food and Drug Administration for application in food products [29], was used at a concentration of 5000 IU per mL (*w*/*v*). The International Unit (IU) is the quantity of nisin required to inhibit a single cell of *Streptococcus agalactiae* in 1000 µL broth medium [30], and 1000 IU of nisin is equivalent to approximately 0.025 mg nisin [14]. The powered nisin was mixed with 2 mL of HEPES buffer and then centrifuged for one minute at 3000 RPM (equivalent to approximately 886 g) using the above-mentioned instrument and rotor to remove the insoluble impurities before filter sterilization [14,31,32]. For carvacrol (Fisher Scientific Company LLC Corp, Hanover Park, IL, USA) and caprylic acid (Fisher Scientific Company LLC Corp.), 0.5% (*v*/*v*) of the antimicrobial (i.e., 7.5 µL antibacterial in 1.5 mL of HEPES buffer) were used in the current study. High-pressure treatments were conducted in PULSE tubes (Pressure BioScience Inc., South Easton, MA, USA) with a total volume of 1.5 mL. The pressure transmission fluid was distilled water (total soluble solids < 30 ppm), and pressure treatments were applied using Hub880 Barocycler unit (Pressure BioScience Inc.). Temperature was precisely regulated by a refrigerated circulating water bath (Model1160s, VWR International, Radnor, PA, USA) connected to stainless steel water jacket surrounding the reaction chamber. The temperature was monitored by a T-type thermocouple (Omega Engineering Inc., Norwalk, CT, USA) inserted inside the wall of chamber and secured using thermal paste (Model 5 AS5-3.5G, Arctic Silver, Visalia, CA, USA), connected to Hub880 Barocycler software (HUB PBI 2.3.11 Software, Pressure BioScience Inc., South Easton, MA, USA) that automatically recorded the temperature and pressure intensity of treatments every three seconds. Trials of the study was conducted under various hydrostatic pressure levels of 350 MPa (with presence of caprylic acid and carvacrol), 450 MPa (at two temperatures of 4 and 40 °C), and at 550 MPa (with the presence of nisin) for up to 7 min. Treatment times exclude the come-up time (<30 s for Hub880 Barocycler) and come-down time (<3 s for Hub880 Barocycler). Our previous study had illustrated that come-up and come-down times of less than one minute have negligible effects (*p* ≥ 0.05) on the microbial reduction capability of pressure-based treatments [33]. As such, reductions obtained in the current study are due to effects of antimicrobials, mild heat, and elevated hydrostatic pressure rather than come-up and come-down times.

### 2.3. Microbiological Enumeration, pH Measurement, and Control of Exposure Time

To assure precise exposure time, each antimicrobial compound was added immediately before the pressure treatment to the PULSE tubes and after homogenization by vortexing, the sample was immediately pressure-treated (<5 s). Additionally, immediately after treatment, samples were neutralized with D/E neutralizing (Difco, Becton Dickinson, Franklin Lakes, NJ, USA) broth (1 mL of treated sample to 3 mL of sterilized D/E broth) to assure neutralization of the antimicrobial compound and assure that antimicrobial residue is not transferred onto surface of the enumeration medium. Thus, the detection limit of the current study is 0.60 log CFU/mL. Samples were additionally placed into ice-water slurry immediately after neutralization to assure temperature exposure times were controlled [12]. After neutralizing the effects of antimicrobials and heat, samples were then 10-fold serially diluted by single strength Maximum Recovery Diluent (MRD, Difco, Becton Dickinson, Franklin Lakes, NJ, USA). To assure further recovery of injured cells, 0.6% of yeast extract was added to Tryptic Soy Agar (TSA, Difco, Becton Dickinson, Franklin Lakes, NJ, USA), the medium used for enumeration of the pathogen. The use of tryptic soy agar with yeast extract (TSA + YE) additionally enhances the recovery of pressure-, heat- and antimicrobial-injured microbial cells and improves external validity of the pressure-based microbiological challenge study [12,34]. The 10-fold diluted samples were spread-plated onto TSA + YE, aerobically incubated at 37 °C for 48 h, and the colony-forming units (CFU) were calculated based on the Bacteriological Analytical Methods (BAM) of the U.S. Food and Drug Administration [35] using a Quebec colony counter. The pH values of the samples were measured using a calibrated digital pH meter prior and after the neutralization (Mettler Toledo AG, Grelfensee, Switzerland).

### 2.4. Experimental Design and Statistical Analyses

This study is summary of four independent experiments: (i) Application of elevated hydrostatic pressure at 450 MPa at 4 and 40 °C; (ii) Application of elevated hydrostatic pressure at 550 MPa and 5000 IU/mL (*w*/*v*) of nisin at 4 °C; (iii) Application of elevated hydrostatic pressure at 350 MPa and 0.5% (*v*/*v*) carvacrol and/or caprylic acid at 25 °C; (iv) Comparison of treatments in the first three experiments with untreated control and the current common food industry standard (treated control). Target inoculation level for experiments 1 and 2 were 7 to 8 and for experiments 3 and 4 were 6 to 7 log CFU/mL. The target inoculations, pressure intensity levels, treatment times and temperatures, and concentrations of antimicrobials were selected based on preliminary trials (data not shown) to assure the microbiological efficacy of the treatments and relevance to stakeholders. Each of the four experiments were conducted in two biologically independent repetitions considered as blocking factor of a randomized complete block design. Each block was additionally consisted of three replications and each replication was obtained by averaging the values of two repetitions as microbiological (instrumental) repetitions. Thus, each represented value is the average of 12 independent observations (two blocks, three replications, two instrumental repetitions). Our previous study indicates that at least five and nine repetitions (for a statistical power of 80% and type I error level of 5%) are needed to observe a 0.15 and 0.10 log difference between two pressure-treated log transformed means as statistically significant, respectively [36].

Data for each trial were separately collected and log-transformed using Microsoft Excel. The inferential statistics were conducted using SAS_9.4_ (SAS Institute Inc., Cary, NC, USA) for conducting analysis of variance (ANOVA) for comparison (Tukey- and/or Dunnett’s-adjusted) of treatments and the controls at type I error level of 5% using the GLM procedures. D-value inactivation index was additionally calculated using reciprocal of slope of the best-fitted linear model by plotting the log-transformed microbial counts as function of treatment time (minutes). The non-linear inactivation index k_max_ was calculated using GInaFiT software (Katholieke Universiteit, version 1.7 Leuven, Belgium).

## 3. Results

### 3.1. Inactivation of S. aureus at 450 MPa of Elevated Hydrostatic Pressure as Affected by Mild Heat

**Figure 1:**
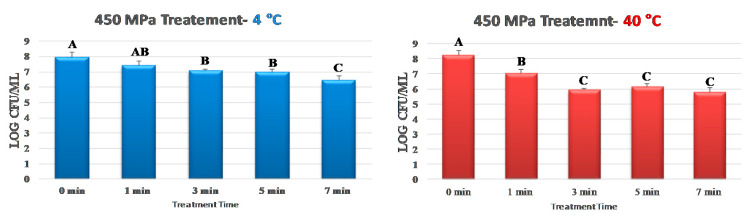
Inactivation of *S. aureus* by elevated hydrostatic pressure at 4 and 40 °C. Values followed by different upper-case letters are statistically (*p* < 0.05) different than each other (pair-wise comparisons, Tukey-adjusted ANOVA).

**Figure 2:**
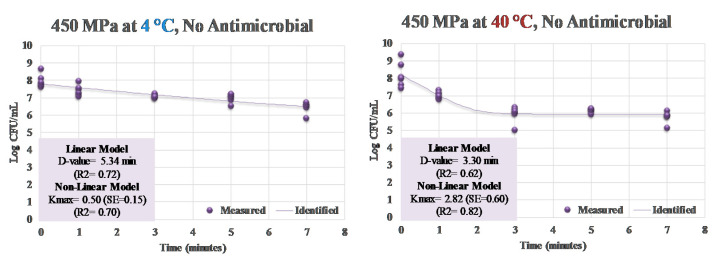
Linear and non-linear inactivation indices for inactivation of *S. aureus* treated by elevated hydrostatic pressure at 450 MPa at 4 and 40 °C with no bacteriocin or bactericidal compound. Measured = Actual observations; Identified = Best-fitted non-linear model.

For samples treated at 4 °C, control counts of *S. aureus* was 7.95 ± 0.4 log CFU/mL (Figure 1). These counts after treatment at 450 MPa were reduced (*p* < 0.05) by 0.54, 0.88, 0.98, and 1.51 log CFU/mL after 1, 3, 5, and 7 min treatments, respectively (Figure 1). The application of mild heat at 40 °C, to a great extent, augmented the decontamination efficacy of the treatment. Control counts and those obtained after 1, 3, 5, and 7 min of treatment were 8.21 ± 0.7, 7.02 ± 0.2, 5.93 ± 0.4, 6.13 ± 0.1, and 5.77 ± 0.3 log CFU/mL, respectively (Figure 1). These reductions (*p* < 0.05) were appreciably higher than those obtained at 4 °C. As an example, while 1.51 log CFU/mL reductions (*p* < 0.05) were obtained after 7 min of treatment at 450 MPa and at 4 °C, the same treatment at 40 °C resulted in 2.44 log CFU/mL reductions (*p* < 0.05) of *S. aureus* (Figure 1). Linear and non-linear inactivation indices exhibited similar trends (Figure 2). While 5.34 min was needed for a 90% reduction in *S. aureus* at 4 °C during a 450-MPa treatment (i.e., D-value = 5.34), the same treatment at 40 °C required 3.30 min for decontamination of the pathogen (Figure 2). The non-linearly calculated K_max_ additionally illustrated the effects of mild heat for enhancing the decontamination efficacy of the treatment. K_max_ values for samples treated at 4 and 40 °C at 450 MPa were 0.50 and 2.81 1/min, respectively (Figure 2). These findings are in harmony with the previous literature, where *S. aureus* was reduced by 1.61 to 2.73 log CFU/mL after 9 min of hydrostatic pressure treatment at 450 MPa [37]. Similarly, 2.69 log reductions in *S. aureus* were reported after pressure treatments of samples at 40 °C and at 400 MPa [38]. 

### 3.2. Inactivation of S. aureus at 550 MPa of Elevated Hydrostatic Pressure as Affected by Nisin

**Figure 3:**
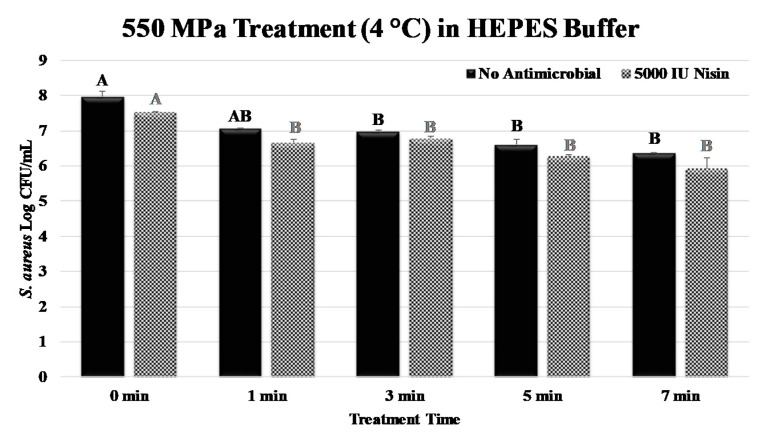
Inactivation of *S. aureus* by elevated hydrostatic pressure (550 MPa at 4 °C) in the presence of 5000 IU of nisin. Values followed by different upper-case letters are statistically (*p* < 0.05) different than each other (pair-wise comparisons, Tukey-adjusted ANOVA). For samples treated without antimicrobial (solid bars) and those treated in the presence of nisin (patterned bars) statistical analyses are presented separately by upper-case letters with different colors.

Prior to the treatment at 500 MPa, counts of *S. aureus* were 7.96 ± 0.2 log CFU/mL (mean ± SE). A one-minute treatment at 550 MPa was not (*p* ≥ 0.05) effective at reducing the pathogen and counts of *S. aureus* were 7.05 ± 0.1 log CFU/mL after the 1 min treatment (Figure 3). The 3, 5, and 7 min treatments were similar to each other (*p* ≥ 0.05) and were effective (*p* < 0.05) in reducing the pathogen. After the treatments, 0.99, 1.36, and 1.61 log CFU/mL reductions (*p* < 0.05) of the pathogen were observed, respectively (Figure 3). Samples treated at 550 MPa of elevated hydrostatic pressure at 4 °C and, in the presence of 5000 IU/mL of nisin, exhibited similar trends. All treatments of 1 to 7 min(s) in the presence of nisin were effective (*p* < 0.05) at reducing the pathogen, and log reductions (*p* < 0.05) for 1, 3, 5, and 7 min treated samples were 0.85, 0.74, 1.25, and 1.59 log CFU/mL, respectively (Figure 3).

**Figure 4:**
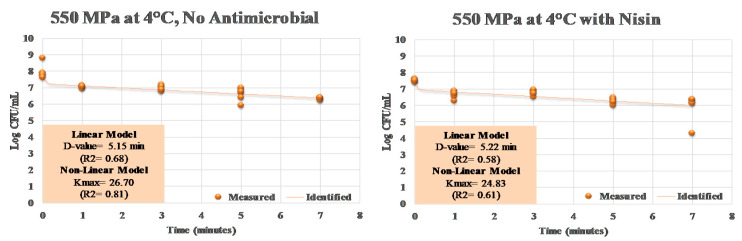
Linear and non-linear inactivation indices for *S. aureus* exposed to elevated hydrostatic pressure (550 MPa at 4 °C) in the presence of 5000 IU of nisin. Measured = Actual observations; Identified = Best-fitted non-linear model.

The calculation of linear and non-linear inactivation indices exhibits a similar trend. D-value, amount of time needed for one log (e.g., 90%) reduction in the pathogen, was similar for elevated hydrostatic pressure treatments with and without added nisin (Figure 4). These values were 5.15 and 5.22 min for samples treated without and with nisin at 4 °C. The inactivation index K_max_ has the unit of 1/min, thus lower values correspond to a higher inactivation rate and vice versa [39]. The K_max_ values for samples treated with and without nisin at 550 MPa and at 4 °C were similar and were 26.70 and 24.83, respectively (Figure 4).

### 3.3. Inactivation of S. aureus at 350 MPa of Elevated Hydrostatic Pressure as Affected by Caprylic Acid and Carvacrol

**Figure 5:**
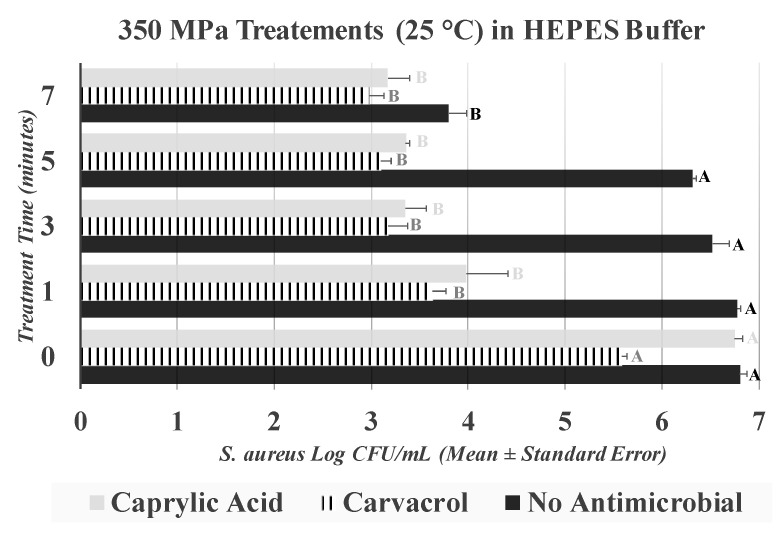
Inactivation of *S. aureus* by elevated hydrostatic pressure in presence of 0.5% (*v*/*v*) caprylic acid and carvacrol. Values followed by different upper-case letters are statistically (*p* < 0.05) different than each other (pair-wise comparisons, Tukey-adjusted ANOVA). For control samples, those treated with caprylic acid, and those treated with carvacrol, statistical analyses are presented separately by upper-case letters with different colors.

This section summarizes the results of samples treated at 25 °C in the presence of 0.5% (*v*/*v*) carvacrol and 0.5% (*v*/*v*) caprylic acid. It is noteworthy that, as previously elaborated, immediately after treatments, samples were neutralized using D/E broth, thus exposure times to antimicrobials were controlled and residual amounts of antimicrobials on surface of the medium were mitigated. For samples treated at 350 MPa (without antimicrobial) the control counts and those obtained after 1, 3, 5, and 7 min of treatments were 6.81 ± 0.1, 6.78 ± 0.0, 6.52 ± 0.2, 6.31 ± 0.0, and 3.80 ± 0.2 log CFU/mL, respectively (Figure 5). These values remained statistically unchanged during the 1, 3, and 5 min of treatment (*p* ≥ 0.05) and only a 7-min treatment was able to reduce (*p* < 0.05) *S. aureus* (Figure 5).

**Figure 6:**
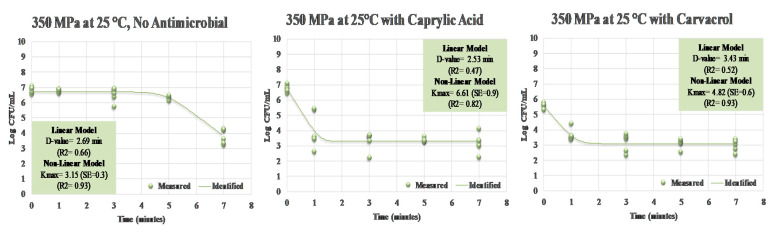
Linear and non-linear inactivation indices for *S. aureus* treated by hydrostatic pressure (350 MPa at 25 °C) in the presence of 0.5% (*v*/*v*) caprylic acid and 0.5% (*v*/*v*) carvacrol. Measured = Actual observations; Identified = Best-fitted non-linear model.

As previously discussed, treatments longer than 5 min are not common in the food industry since high-pressure processing is a batch system and longer treatment times could reduce the efficiency of production [12,13,14,16]. The application of carvacrol (*p* < 0.05) was effective to enhance the decontamination efficacy of treatments with ≤ 5 min of duration. Specifically, after 1, 3, and 5 min of treatment at 350 MPa in presence of 0.5% carvacrol, counts of the pathogen were reduced (*p* < 0.05) by 1.95, 2.41, and 2.48 log CFU/mL, respectively (Figure 5). The corresponding reductions for the same treatment without any antimicrobials were 0.03, 0.28, and 0.49, respectively, indicating that the addition of carvacrol could, to a great extent, augment the efficacy of pressure pasteurization, particularly for short-term treatments of ≤ 5 min. Caprylic acid at 0.5% was also efficacious (*p* < 0.05) for enhancing the decontamination efficacy of the treatments. Counts of treated samples at 350 MPa in the presence of caprylic acid were reduced (*p* < 0.05) by 2.77, 3.40, 3.39, and 3.58 log CFU/mL after 1, 3, 5, and 7 min of treatment (Figure 5). It is important to note that, for a long-term treatment of more than 5 min, the efficacy of both caprylic acid and carvacrol faded and log reductions were similar to the samples treated without any antimicrobial. Thus, our results illustrate that the tested antimicrobials are efficacious for short-term treatments, such as those that are currently the common processing parameters in the private food industry. Inactivation indices illustrate similar trends, with the D-value, and the K_max_ of all three treatments of up to 7 min remain nearly identical (Figure 6).

### 3.4. Summary of Inactivation Efficacy of Treatments

**Figure 7:**
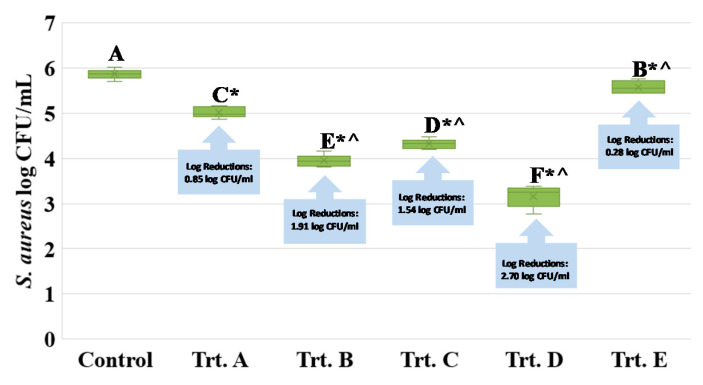
Inactivation of *S. aureus* by elevated hydrostatic pressure in presence of bacteriocin and bactericidal compounds and mild heat. Control: Untreated control (0 MPa at 4 °C for 3 min); Trt. A: Treated control (650 MPa at 4 °C for 3 min). This treatment is currently the industry standard for pressure-based pasteurization [12,13,14]. Trt. B: Treatment of 550 MPa at 4 °C for 3 min with 5000 (w/v) IU nisin. This treatment is further investigated under the second experiment of this study, summarized in Figure 3 and Figure 4. Trt. C: Treatment of 450 MPa at 40 °C for 3 min. This treatment is further investigated under the first experiment of the study, summarized in Figure 1 and Figure 2. Trt. D: Treatment of 350 MPa at 25 °C for 3 min with 0.5% (v/v) carvacrol. Trt. E: Treatment of 350 MPa at 25 °C for 3 min with 0.5% (v/v) caprylic acid. The last two treatments are further investigated under the third experiment of the study, summarized in Figure 5 and Figure 6. Values followed by different upper-case letters are statistically (*p* < 0.05) different than each other (pair-wise comparisons, Tukey-adjusted ANOVA). Values followed by * are statistically (*p* < 0.05) different than untreated control (Dunnett’s-adjusted ANOVA). Values followed by ^ are statistically (*p* < 0.05) different than Trt. A., e.g., treated control (Dunnett’s-adjusted ANOVA).

Currently, the food industry relies on treatments of 650 MPa, commonly lasting for 3 min, for pressure-based pasteurization of various products [12,13,14]. As previously discussed, one of the main challenges for the production of pressure-treated commodities is slightly higher operation costs relative to traditional heat-treated products. Thus, this study investigated the application of lower intensities of pressure augmented with GRAS-listed bacteriocin and bactericidal compounds. This synergism between elevated hydrostatic pressure and antimicrobials could reduce the operation cost and thus enhance the competitiveness of pressure-treated commodities. Additionally, it could provide protection for products during shelf-life due to the residual effects of antimicrobials [12,13,14]. This experiment, summarized in Figure 7, was conducted to compare the treatments from experiments 1 to 3 to the common industry standard (Trt. A) and to an untreated control (Control). This common treatment from the food industry (650 MPa at 4 °C, for 3 min) resulted in a 0.85 log CFU/mL reduction of the pathogen (Figure 7). The 3-min treatments at 550 MPa (at 4 °C) and with 5000 (*w*/*v*) IU nisin (Trt. B), 450 MPa at 40 °C (Trt. C), and 350 MPa (at 25 °C) and 0.5% (*v*/*v*) carvacrol (Trt. D), were more effective (*p* < 0.05) for the inactivation of *S. aureus*, compared to Trt. A (Figure 7). Log reductions (*p* < 0.05) for these three treatments were 1.91, 1.54, and 2.70 log CFU/mL, respectively (Figure 7).

## 4. Discussion

It is important to note that, although the pathogen was reduced by up to > 90% due to treatment with 550 MPa of elevated hydrostatic pressure with and without 5000 IU/mL of nisin, treatments left behind as much as >5 log CFU/mL of the pathogen, indicating that pressure at this intensity with or without nisin is not solely sufficient to assure the safety of a product, and requires additional decontamination hurdle(s). Thus, this level of nisin and pressure could be incorporated only as one of the hurdles in a multiple hurdle technology [40], not as the sole antimicrobial treatment.

It is also noteworthy that the utilization of the HEPES buffer is common in the high-pressure processing literature, since the medium could maintain a buffered environment under the elevated hydrostatic pressure [34]. Thus, the reductions obtained in the current study could be attributed to the effects of elevated hydrostatic pressure, heat, and the antimicrobials rather than any intrinsic factor of the medium [12]. The application of nisin was reported to be efficacious to augment the pressure-based pasteurization of bacterial pathogens in the past. Nisin was able to significantly increase the decontamination effectiveness of a pressure-based treatment against a Gram-positive bacterium, *Listeria monocytogenes* [14]. Other studies indicate an even lower concentration of nisin, such as 100 IU/mL, could enhance the decontamination efficacy of high-pressure pasteurization against pathogenic organisms such as *Escherichia coli*, *Salmonella* Enteritidis, *Salmonella* Typhimurium, *Shigella sonnei*, *Shigella flexneri*, *Pseudomonas fluorescens,* and *S. aureus* [41]. Similarly, nisin was efficacious to enhance the decontamination efficacy of other non-thermal treatments, such as high-intensity pulsed electric fields for the elimination of *S. aureus* [42]. *Staphylococcus carnosus* was similarly reduced by synergism of 500 MPa of pressure and nisin, indicating that other species of *Staphylococcus* could additionally be decontaminated using a combination of elevated hydrostatic pressure and nisin [43]. These synergistic effects from the literature were typically obtained for treatments of as long as 30 min. In food processing, treatments longer than 5 min are not common and the vast majority of current commercial treatments are limited to 3 min [12,13,14,16]. Our results indicate while this antimicrobial is very effective against other Gram-positive pathogens such as *Listeria monocytogenes* [15], the application of nisin for time intervals of less than 10 min and at 4 °C only has mild antibacterial benefits against *S. aureus* (Figure 3).

To assimilate the effects of mild heat for augmenting the decontamination efficacy of pressure-based pasteurization, the experiment was conducted at two controlled temperatures of 4 and 40 °C. It is noteworthy that a pressure of 450 MPa is more than four times higher than the pressure in Mariana Trench, the earth’s deepest oceanic trench [44]. However, this pressure is still considered as a medium level of pressure in food manufacturing, as vast majority of pressure-treated products in commerce are treated at 650 MPa [12,13,14,16]. Currently, pressure-treated products typically have slightly higher operation cost relative to traditional thermally treated products, thus the application of lower levels of pressure coupled with mild heat and/or bacteriocin and bactericidal compounds could be of great importance for stakeholders to optimize their cost. The application of pressure at higher levels is typically associated with a higher cost of operation and maintenance of the pressure vessels [12].

This finding is in harmony with the previous literature, where it was shown that antimicrobials known to be effective against Gram-positive organisms such as *Listeria monocytogenes* are capable of enhancing the decontamination efficacy of elevated hydrostatic pressure for short-term treatments, and the efficacy of the antimicrobial faded as treatment time increased [14]. Studies investigating the synergism of elevated hydrostatic pressure, carvacrol, and caprylic acid for the inactivation of *S. aureus* is very limited in the literature. However, the positive effects of carvacrol and caprylic acid for enhancing pressure-based inactivation of Gram-positive organisms, such as *Listeria monocytogenes*, and Gram-negative bacteria, such as Shiga toxin-producing *Escherichia coli*, were reported in the literature [13,45]. It is noteworthy that although infections with *S. aureus* are not as fatal as the vast majority of the main foodborne diseases [2], this pathogen is highly prevalent among food workers, and thus additional studies on the decontamination of this microorganism could be of great importance to the private food industry. One study conducted in the fish-processing factory showed that as many as 62% of the workers in one operation tested positive for *S. aureus* [8]. Other studies indicate that > 26% of food handlers could be a carrier of this pathogen [9]. As previously articulated, this study was conducted in the HEPES buffer, capable of maintaining a steady pH under the elevated hydrostatic pressure. Thus, the reductions could be solely attributed to the effects of temperature, the antimicrobials and/or elevated hydrostatic pressure. Future studies could further explore the effects of various intrinsic and extrinsic factors, particularly pH, in various food vehicles to identify and optimize the use of various bacteriocin and bactericidal compounds for improving the microbiological safety and cost-effectiveness of pressure-based treatments.

This study investigated the effects of three bacteriocin and bactericidal compounds (carvacrol, caprylic acid, and nisin), that are part of the generally recognized as safe (GRAS) list of the U.S. Food and Drug Administration as food additives [17]. Antibiotics efficacious for the treatment of *S. aureus* infections such as oxacillin, methicillin, or cefoxitin could also be used to augment the decontamination efficacy of elevated hydrostatic pressure to reduce this pathogen in the food chain in future experiments. These antibiotics, if granted GRAS status, could additionally become part of food formulations for the control of this pathogen. In addition, *S. aureus* is among the 11 pathogens that are considered “serious threats” by the U.S. Centers for Disease Control and Prevention for development of antibiotic resistance [46]. Thus, future studies comparing the susceptible and drug-resistant (such as methicillin-resistant *S. aureus*) phenotypes of this pathogen could be projects of great importance for continuation of the experiments articulated in our study. Finally, it is noteworthy that *S. aureus* is a versatile and dangerous pathogen that, in addition to causing systematic infection, could secrete exotoxins that could be relatively stable in the food environment [47]. The current study investigated the effects of the proposed treatments against the planktonic cells of the pathogen to prevent infections and encoding of secondary metabolites such as exotoxins. Studies exploring the pressure sensitivity of *S. aureus* exotoxins could be novel and very important follow-up experiments for future researchers.

## 5. Conclusions

Under the condition of our experiments, we observed that nisin had only minor effects in augmenting the decontamination efficacy of the pressure-based (550 MPa) pasteurization of *S. aureus*. Carvacrol and caprylic acid, however, were able to greatly enhance the decontamination efficacy of elevated hydrostatic pressure at 350 MPa and at 25 °C for treatments shorter than 5 min. Mild heat (40 °C) was similarly effective to increase the decontamination efficacy of pressure-based treatments for inactivation of *S. aureus.*

These findings indicate that practitioners of this technology could benefit from the synergism of mild heat, and bacteriocin and/or bactericidal compounds to assure the microbial safety of products while utilizing moderate levels of hydrostatic pressure. This approach optimizes the cost of operation and the competitiveness of pressure-treated products, since extreme levels of pressure are typically associated with higher operation and maintenance cost. Additionally, this could lead to co-benefits such as the potential for the better retention of nutrients and bioactive compounds and better product shelf-life due to the residual effects of antimicrobials in product formulation. Our results additionally illustrate that the product, pathogen, level of elevated hydrostatic pressure, antimicrobial, and temperature would need to be carefully considered in a hurdle microbiological validation study to ensure that selected intrinsic and extrinsic factors are capable of assuring the microbiological safety of the product and its economic feasibility.
